# Serum Proteomic Analysis Reveals Vitamin D-Binding Protein (VDBP) as a Potential Biomarker for Low Bone Mineral Density in Mexican Postmenopausal Women

**DOI:** 10.3390/nu11122853

**Published:** 2019-11-21

**Authors:** Mayeli M. Martínez-Aguilar, Diana I. Aparicio-Bautista, Eric G. Ramírez-Salazar, Juan P. Reyes-Grajeda, Aldo H. De la Cruz-Montoya, Bárbara Antuna-Puente, Alberto Hidalgo-Bravo, Berenice Rivera-Paredez, Paula Ramírez-Palacios, Manuel Quiterio, Margarita Valdés-Flores, Jorge Salmerón, Rafael Velázquez-Cruz

**Affiliations:** 1 Laboratorio de Genómica del metabolismo óseo, Instituto Nacional de Medicina Genomica (INMEGEN), Ciudad de México 14610, Mexico; mmaye.sol@gmail.com (M.M.M.-A.); eramirez@inmegen.gob.mx (E.G.R.-S.); audelacm@gmail.com (A.H.D.l.C.-M.); 2 Subdirección de Aplicaciones Clínicas, Instituto Nacional de Medicina Genómica (INMEGEN), Ciudad de México 14610, Mexico; daparicio@inmegen.gob.mx (D.I.A.-B.); jreyes@inmegen.gob.mx (J.P.R.-G.); 3 Consejo Nacional de Ciencia y Tecnología (CONACYT)-Laboratorio de Genómica del Metabolismo Óseo, Instituto Nacional de Medicina Genómica (INMEGEN), Ciudad de México 14610, Mexico; 4 Laboratorio de Enfermedades Cardiovasculares, Instituto Nacional de Medicina Genómica (INMEGEN), Ciudad de México 14610, Mexico; bapuente@gmail.com; 5 Laboratorio de Genética, Dirección de Investigación, Instituto Nacional de Rehabilitación, Ciudad de México 14389, Mexico; dr_genetica@yahoo.com (A.H.-B.); marvaldes@yahoo.com (M.V.-F.); 6 Centro de Investigación en Políticas, Población y Salud, Facultad de Medicina, Universidad Nacional Autónoma de México, Ciudad de México 04510, Mexico; bereriveraparedez7@gmail.com (B.R.-P.); jorge.salmec@gmail.com (J.S.); 7 Unidad de Investigación Epidemiológica y en Servicios de Salud, Instituto Mexicano del Seguro Social (IMSS), Cuernavaca Morelos 62000, Mexico; paula_rzps@hotmail.com; 8 Centro de Investigación en Salud Poblacional, Instituto Nacional de Salud Pública, Cuernavaca, Morelos 62100, Mexico; mquiterio@insp.mx

**Keywords:** biomarker, bone mineral density, osteoporosis, proteomics, vitamin D-binding protein (VDBP)

## Abstract

Osteoporosis is a skeletal disease mainly affecting women over 50 years old and it represents a serious public health problem because of the high socioeconomic burden. This disease is characterized by deterioration of bone microarchitecture, low bone mineral density (BMD), and increased risk of fragility fractures. This study aimed to identify serum useful proteins as biomarkers for the diagnosis and/or prognosis of osteoporosis and fracture risk. We collected 446 serum samples from postmenopausal women aged ≥45 years old. Based on the BMD measurement, we classified the participants into three groups: osteoporotic, osteopenic, and normal. In an initial discovery stage, we conducted a proteomic approach using two-dimensional differential gel electrophoresis (2D-DIGE). The peptides into the spots of interest were identified through matrix-assisted laser desorption/ionization-time of flight (MALDI-TOF/TOF). Enzyme-linked immunosorbent assay (ELISA) was performed to validate the proteins of interest. We identified 27 spots of interest when comparing low BMD versus normal BMD postmenopausal women. Based on their relevance in bone metabolism, we analyzed three proteins: ceruloplasmin (CP), gelsolin (GSN), and vitamin D-binding protein (VDBP). Our results demonstrated that low serum VDBP levels correlate with low BMD (osteopenic and osteoporotic). Therefore, VDBP could be considered as a novel, potential, and non-invasive biomarker for the early detection of osteoporosis.

## 1. Introduction

Osteoporosis is a skeletal disorder characterized by a decrease in bone mass and microarchitecture deterioration. Osteoporosis results in an increased risk of fragility fractures [1]. Osteopenia is an early stage of osteoporosis and it is asymptomatic [2,3]. Fragility fractures are the most serious complications of osteoporosis. Among them, hip fracture represents an important cause of loss of independence. The most affected population is postmenopausal women (>50 years old) and hip fracture is the main reason for morbidity and mortality [4]. In Mexico, approximately 1 of every 12 women will suffer a fracture after the age of 50. This number is likely to rise due to the increase in life expectancy and population aging [5,6].

The measurement of bone mineral density (BMD) using a dual-energy X-ray absorptiometry (DXA) scan is the gold standard for osteoporosis diagnosis [7]. However, about 50% of postmenopausal women who had presented an osteoporotic fracture have normal BMD [8]. This method has some limitations: low BMD is a risk factor for fracture, not a disease marker, as it reflects the present status of bone. Another available resource for estimating fracture risk is the fracture risk assessment tool (FRAX). FRAX considers risk factors like gender, age, body mass index (BMI), and BMD to estimate the odds of suffering a fragility fracture. [9] However, FRAX has a limited accuracy [10], it does not incorporate risk factors such as those associated with falls, markers of bone turnover, low calcium intake, and vitamin D status [11]. 

The serum is one of the most studied biological fluids. There are several serum molecules, related to specific physiological or pathological conditions [12]. Some studies using serum from osteoporotic women have suggested osteocalcin, alkaline phosphatase (ALP), carboxy- and amino-terminal propeptide of type I collagen (P1NP, P1CP), cathepsin K, carboxi-terminal telopeptide (CTX), amino-terminal telopeptide (NTX), matrix metalloproteinase (MMP)-generated (CTX-MMP or ICTP), and type I collagen fragments, as bone resorption and formation biomarkers [13,14].

However, all these biochemical markers are not fully specific or sensitive as early-stage markers of osteoporosis. Therefore, these biochemical markers do not meet the requirements to be used as specific non-invasive markers for osteoporosis or fracture risk prediction in clinical practice. In order to detect osteoporosis and osteoporotic fracture at early stages, it is necessary to identify biomarkers for those asymptomatic women who may develop osteoporosis. Biomarkers could be serum proteins, metabolites, or electrolytes whose differential expression indicates the presence of disease. Therefore, the search for novel biomarkers for osteoporosis is essential and will be beneficial for evaluation and treatment for osteoporosis patients, particularly at the early stages.

To date, most studies for the discovery of bone remodeling markers have focused on the identification of fragments derived from individual proteins [15]. However, these proteins have not been validated because of their great variability, both analytical and biological. Several factors, such as fracture repair, age, renal, hepatic function, menopausal status, and the association with other diseases, are influencing the variability [11,13]. 

Serum proteomic analysis has emerged as an important approach for biomarker discovery of numerous diseases, it can also provide information about the underlying pathological processes [16]. Proteomics has made valuable contributions to understand the physiopathology of osteoporosis and to identify potential biomarkers for diagnosis and prognosis [17]. Therefore, these developments are important as a first step towards the identification of novel non-invasive biomarkers. This knowledge will allow the development of new screening strategies for detecting the population at risk and, therefore, mitigating the social and economic burden. 

In the present study, we performed two-dimensional differential gel electrophoresis (2D-DIGE) analyses conjugated with matrix-assisted laser desorption/ionization-time of flight (MALDI-TOF/TOF) to analyze serum derived from postmenopausal women with normal and low BMD (osteopenia/osteoporosis) to identify proteins related to bone metabolism that could be used as new biomarkers for the diagnosis and prognosis of osteoporosis and fracture risk.

## 2. Materials and Methods

### 2.1. Study Participants

We collected serum samples from 425 unrelated postmenopausal women. This group represents a sub-sample of participants of “The Health Workers Cohort Study (HWCS)”, from the Mexican Social Security Institute (IMSS). The HWCS is a long-term study of workers from the IMSS in the city of Cuernavaca, Morelos, that focuses on lifestyle and the development of chronic diseases [18]. All women included in this study had Mexican-Mestizo origin. Samples were collected in a second assessment from 2010 to 2013. The detailed characteristics of the postmenopausal women were previously reported [19].

A subgroup of 30 postmenopausal women (10 with normal BMD, 10 with osteopenia (OS), and 10 with osteoporosis (OP)), was selected for the 2D-DIGE as an initial discovery analysis (Table 1). Women were selected based on the following inclusion criteria: ≥45 years of age, postmenopausal status (defined as 12 consecutive months without menstruation), and available BMD measurements. Women with diabetes mellitus, chronic liver diseases, rheumatoid arthritis, collagen diseases, and those under corticosteroids, anticonvulsants, bisphosphonates, or hormone replacement therapy, were excluded from the analysis. Additionally, an independent group of 21 postmenopausal women >50 years old, with the diagnosis of osteoporosis or fragility fractures from the National Institute of Rehabilitation, was used for validation of candidate proteins by enzyme-linked immunosorbent assay (ELISA). Fracture sites were as follows: 17 at the femoral neck, 3 at the wrist, and 1 at the lumbar spine. The protocol was approved by the Mexican Social Security Institute (No. 12CEI 09 006 14), National Institute of Rehabilitation (56/13-C01-69706), and the National Institute of Genomic Medicine (314-07/2017/I), following the Declaration of Helsinki (13/LO/0078). All participants provided written informed consent. 

### 2.2. Bone Mineral Density (BMD) Measurement and Osteoporosis Diagnosis

Hip BMD (g/cm^2^) was determined using a Lunar DPX NT dual-energy X-ray absorptiometry (DXA) instrument (Lunar Radiation Corp., Madison, WI, USA). Subjects were categorized into three groups according to their hip T-score. The normal group (NOR) included women with T-scores from −1.0 to +1, the osteopenic (OS) group included women with T-scores from −1.0 to −2.5, and the osteoporotic (OP) group included women with T-scores from −2.5 to −4. 

### 2.3. Serum Sample Preparation 

Blood samples were collected after 8 h of fasting. Serum was obtained and stored at −70 °C until use. Albumin and IgG were depleted using albumin and IgG cartridge to increase the scope in the detection of spots (Qiagen, Hilden, North Rhine-Westphalia, Germany) on a high-performance liquid chromatography (HPLC) equipment (Waters Alliance 2695, Inc., Milford, MA, USA) following the manufacturer’s instructions (Figure S1 and see supplementary material for further details). After depletion, quantification of protein was performed using a 2D Quant kit (GE Healthcare, Chicago, IL, USA), following the manufacturer’s instructions.

### 2.4. 2D DIGE and Image Analysis

Five samples from each group were pooled to make two pools for each condition, in total there were 10 samples from each group, i.e., normal, osteopenia, and osteoporosis were used for an initial discovery stage. Each pool was quantified and labeled according to CyDye DIGE minimal protocol (GE Healthcare, Chicago, IL, USA). Briefly, 50 µg of protein from each pool were labeled using 400 pmol of fluorescent cyanine dyes. Besides, an internal standard containing the same amount of protein was labeled with Cy2 (Table S2). The internal standard was run on all the DIGE gels to assess the reproducibility and minimize gel-to-gel variation. 

First-dimension isoelectric focusing (IEF) was done on immobilized pH gradient (IPG) strips 4–7, 24 cm (Bio-Rad), rehydration was carried out overnight at room temperature. After rehydration, isoelectric focusing was performed with an Ettan IPGphor 3 system (GE Healthcare). The IPG strips were then equilibrated in equilibration buffer (80 mM dithiothreitol and 169 mM iodoacetamide) for 15 min. After equilibration, proteins were separated in the second dimension by sodium dodecyl sulfate–polyacrylamide gel electrophoresis (SDS-PAGE) using 12% gels on an Ettan DALT-Twelve electrophoresis system (GE Healthcare) and directly scanned using the Ettan DIGE Imager (GE Healthcare). For differential expression analysis, the images were normalized and analyzed using the DeCyder 2D software (V 6.5) (GE Healthcare), see supplementary material for further details. To identify significant changes in protein expression between experimental groups, we used the differential in-gel analysis (DIA) module followed by the biological variation analysis (BVA) module. For the analyses, each Cy3 or Cy5 gel image was assigned to an experimental condition, either control, osteopenic, or osteoporotic, and all Cy2 images were classified as internal standards. The gel with the highest spot count was assigned as the master gel. Matching between gels was performed utilizing the in-gel standard in each image pair. Landmarking and manually confirming potential spots of interest further improved matching. Statistical analysis was performed in “protein table mode” in the BVA module, it included the student’s *t*-test, analysis of variance (ANOVA), fold change calculation, and false discovery rate (FDR) adjustment. FDR aims to achieve an acceptable ratio of true and false positives. An FDR rate of 5% means that, on average, 5% of changes identified as significant would be expected to have arisen from type-one errors [20]. The student’s *t*-test was performed for every matched spot-set, comparing the average and standard deviation of protein abundance for a given spot. Statistical significance was considered when fold change values were ≥1.5 and *p* values ≤ 0.05.

### 2.5. Protein Identification by MALDI TOF/TOF Mass Spectrometry

The spots of interest were manually excised from the gels and dried at room temperature with 100% acetonitrile (ACN) for 5 min. Thereafter, proteins were cleaved using mass spectrometry grade trypsin to produce tryptic peptides (Promega, Madison, WI, USA). In-gel digestion was initiated by the addition of trypsin (20 µg/µL) buffer for 1 h at 4 °C, afterwards, the suspension was incubated overnight at 37 °C. Isolated peptides were centrifuged and reconstituted with 5% formic acid/50% ACN. A ZipTip pipette tip containing C18 resin (Millipore, Billerica, MA, USA) was used for clearance of chemical reagents and eluted with 50% ACN/0.1% Trifluoroacetic Acid (Sigma-Aldrich, St. Louis, MO, USA). Spectra were acquired using a 4800 MALDI-TOF/TOF Analyzer (Applied Biosystems/AB Sciex, Waltham, MA, USA), see supplementary material for further details. Protein identification was performed by peptide mass fingerprinting using the ProteinPilot software version 2.0 (AB Sciex, Framingham, MA, USA) with the built-in Paragon algorithm as the search engine. Results of MS/MS were compared against “Homo sapiens” species using the UniProt database.

### 2.6. ELISA Analysis

Serum VDBP, CP, and GSN protein levels from the postmenopausal women analyzed in the initial discovery stage (*n* = 10, per group) and 44 more women from the HWCS, were assessed by ELISA using a commercial kit (Cat No. DBDBP0B, R&D Systems, Inc., Minneapolis, MN, USA, Cat No. E-EL-H0152 and E-EL-H1786, Elabscience Biotechnology Co., Ltd. Houston, TX, USA, respectively), following the manufacturers’ instructions. As VDBP significantly discriminates between normal, osteopenia, and osteoporosis groups, we performed a validation analysis of the remaining serum samples (*n* = 395) from the HWCS, to complete a total of 425 samples. VDBP was also assessed in the women with fragility fractures (*n* = 21). 

### 2.7. Statistical Analysis

Analyses of clinical variables between study groups were carried out through ANOVA or the Dunn test. Protein levels from ELISA analysis were calculated by one-way ANOVA or Dunnett’s/Dunn’s multiple comparisons test in GraphPad Prism 5 (GraphPad Software, Inc. San Diego, CA, USA). We performed a logistic regression model for osteopenia/osteoporosis and the potential biomarkers (VDBP, CP and GSN), adjusted by age and body mass index, to generate a predictor of the model by which we estimated the receiver operating characteristic (ROC) curve. The receiver operating characteristic curve (ROC) was calculated and a cutoff value that best discriminated women with low BMD (osteopenia/osteoporosis) from normal postmenopausal women was obtained. Sensitivity, specificity, positive predictive value (PPV), and negative predictive value (NPV) were estimated with a confidence interval of 95%. A *p*-value <0.05 was considered statistically significant. The statistical analyses were performed using Stata version 13.0 software (StataCorp LP, College Station, TX, USA).

## 3. Results

### 3.1. 2D-DIGE Differential Protein Expression Analysis 

**Figure 1:**
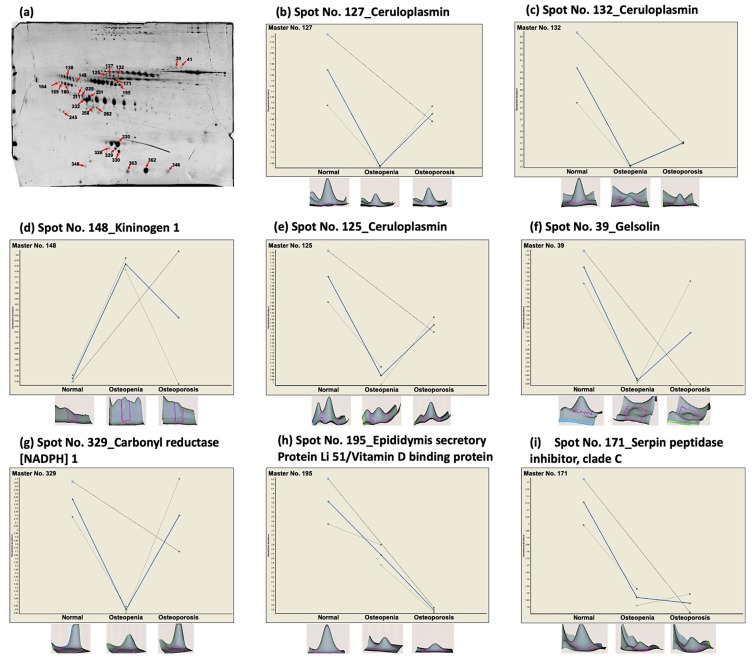
Differentially expressed proteins in serum derived from postmenopausal women, identified by 2D-DIGE analyses. (**a**) A representative preparative 2D gel of the proteins derived from serum of postmenopausal women, normal, osteopenia, and osteoporosis, stained with Coomassie blue. Arrows and numbers mark the spots with differential expression. (**b**–**i**) Three-dimensional (3D) images and graphical representation of selected serum proteins with statistically significant (*p* < 0.05) differential expression when comparing osteopenia or osteoporosis women to the normal group. Data are represented as mean ± standard deviation (SD), graphs show the decrease/increase in the standardized log abundance of spot intensity in the groups of study.

After the 2D-DIGE analysis, a total of 365 spots were detected across all three analyzed groups (data not shown). Protein profiles were compared between the OS or OP groups versus the NOR group. When comparing OP versus NOR through differential in-gel analysis (DIA), we identified 109 differentially expressed spots based on a fold change of ≥1.5, but only 6 spots reached statistical significance (*p* ≤ 0.05). In contrast, a total of 120 spots had a ≥1.5-fold change difference between OS and NOR, but only 28 spots reached statistical significance (*p* ≤ 0.05). We also performed a comparison between OP versus OS, identifying 59 spots with fold change ≥1.5, from these, 5 spots had a significant *p*-value. Furthermore, we observed differences among the study groups for multiple spots from some proteins that did not reach statistical significance. Thus, in an attempt to identify proteins related to BMD variation, additional spots were included in the MALDI-TOF/TOF analysis, based only on their fold change. Additionally, with this strategy, we identified a total of 28 spots in the SDS-PAGE gels through pairwise comparisons of the three studied groups (Figure 1a). 

### 3.2. MALDI-TOF/TOF Protein Identification Analysis

To identify the proteins present in the 28 differentially expressed spots from the 2D-DIGE analyses, the spots were manually excised for mass spectrometry analysis. We successfully identified the peptides present in 27 of the 28 spots by MALDI-TOF/TOF. The remaining spot produced virtually empty spectra, probably due to its small amount of protein; therefore, no peptides could be identified. The protein-pilot software was used to interrogate the UniProt database. Supplementary Table S3 summarizes the 27 proteins identified and the fold change observed when comparing postmenopausal women with low BMD versus normal BMD. The ID Match in the table corresponds to those indicated in the preparative 2-D PAGE gel shown in Figure 1a. 

We identified 16 down-regulated and 11 up-regulated proteins (Table S3 and peptides identification in Table S4). The 27 spots represented a total of 12 different proteins. Some proteins were present in more than one spot. We found that six proteins met the selection parameters (fold change ≥ 1.5 and *p*-value ≤ 0.05): ceruloplasmin (CP), kininogen 1 (KNG1), gelsolin (GSN), carbonyl reductase (NADPH) 1 (CBR1), epididymis secretory protein Li 51 (HEL-S 51), and serpin peptidase inhibitor C (SERPINC1). HEL-S 51 (also known as vitamin D-binding protein, VDBP) was identified with the lowest fold change, on average −1.9, and the highest protein coverage (Table 2). 

We conducted a comprehensive literature review in the Medline database for these six proteins, to investigate their relevance regarding bone metabolism and their potential use as biomarkers. The proteins GSN, CP, and VDBP have previously been related to bone metabolism and proposed as potential biomarkers to identify BMD variation [21,22,23,24,25,26,27]. Additionally, we included other proteins with a role in bone remodeling or metabolism, as described in previous studies, such as alpha-2-S-glycoprotein (AHSG) [28,29], alpha-1-antitrypsin (SERPINA1) [30], and haptoglobin (HP) [31]. These proteins showed ≤1.5-fold change; however, they did not reach statistical significance (Supplementary Table S3 and Supplementary Figure S2). Finally, the three proteins fulfilling the fold change and *p*-value criteria (Figure 1b–i) and with relevance, according to the literature, were GSN, VDBP, and CP. These proteins were selected for validation analysis. 

### 3.3. Gelsolin (GSN), Vitamin D-Binding Protein (VDBP), Ceruloplasmin (CP), and ELISA Validation

**Figure 2:**
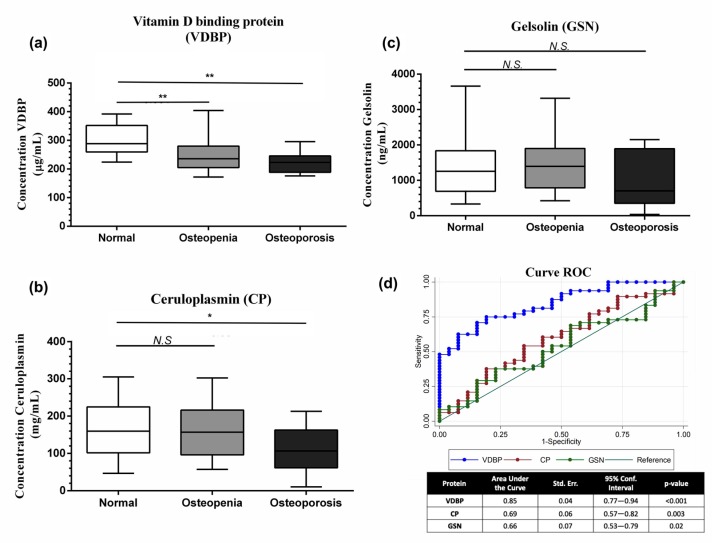
Validation as a potential biomarker. Box-and-whisker plot showing the serum levels of (**a**) vitamin D-binding protein (VDBP), (**b**) ceruloplasmin (CP), and (**c**) gelsolin (GSN) in the Normal, Osteopenia, and Osteoporosis groups after the validation through ELISA. (**d**) Receiver operating characteristic (ROC) analysis of the three candidate proteins useful as biomarkers. * *p* < 0.05, ** *p* < 0.001, N.S Not statistically significant, compared to normal group.

Based on the results obtained through proteomic analysis (initial discovery stage), we decided to quantify VDBP, GSN, and CP proteins by ELISA (NOR: *n* = 26, OS: *n* = 29, OP: *n* = 19) (Table 1). VDBP serum levels in the OS and OP groups were significantly lower than the levels of the NOR group (*p* < 0.001) (Figure 2a). CP serum levels were lower in the osteoporotic group compared to normal (*p* = 0.03) (Figure 2b). There was no difference in GSN serum levels between the three study groups (Figure 2c). 

ROC-curve analysis showed an AUC for VDBP = 0.85 (95% confidence interval (CI), 0.77–0.94, *p* = < 0.001), CP = 0.69 (95% CI, 0.57–0.82, *p* = 0.003), and GSN = 0.66 (95% CI, 0.53–0.79, *p* = 0.02) (Figure 2d). On the other hand, after merging VDBP with CP and VDBP with GSN, the AUC values were 0.848 and 0.855, respectively. Furthermore, the analysis showed that VDBP plus CP and GSN did not improve the AUC value (0.85 (95% CI, 0.76–0.94, *p* = 0.001)), data not shown.

### 3.4. Serum VDBP Levels as a Potential Biomarker

**Figure 3:**
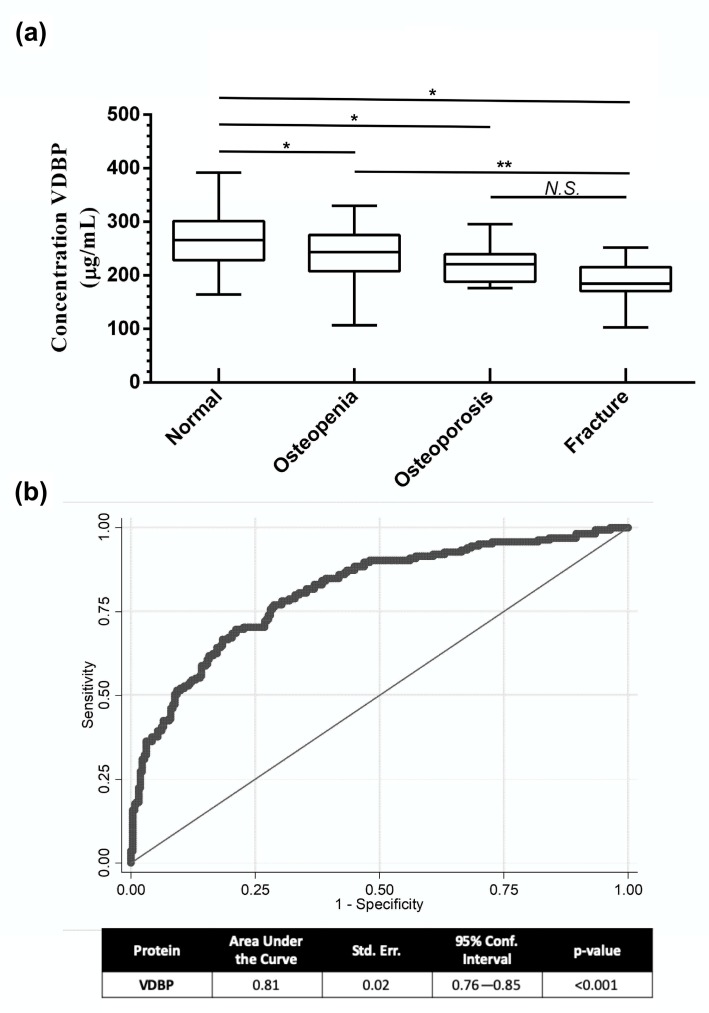
Serum levels of VDBP as a biomarker for low BMD. (**a**) Serum levels of VDBP measured by ELISA in the samples from the normal, Osteopenia, Osteoporosis, and fracture groups. (**b**) The ROC curve was plotted to illustrate the performance of the candidate biomarker (VDBP) for the detection of individuals with low BMD. The area under the curve (AUC), standard error, confidence interval (CI), and *p*-value are shown in the table at the bottom of the graph. * *p* < 0.001 compared to normal group, ** *p* < 0.001 compared to osteopenia group, N.S: Not statistically significant, compared to osteoporosis group.

Based on the differential expression of VDBP and the results of the ROC analysis, we selected VDBP for validation as a potential biomarker in all the postmenopausal women from the HWCS (*n* = 425). In addition, an independent group of patients with fragility fracture was included in the validation analysis (*n* = 21) (Table S1). A significant difference between the groups was observed (*p* < 0.001) (Figure 3a). Moreover, we observed a significant positive correlation between VDBP levels and BMD (*r* = 0.23, *p* < 0.001). We generated a predictor of the model to estimate the sensitivity and specificity of the combination of these variables. ROC analysis showed an AUC of 0.81 (95% CI, 0.76–0.85, *p* < 0.001), sensibility of 70%, and specificity of 75%, with a cutoff of 260 µg/mL (Figure 3b). Fracture samples were not included in this model because BMD values were not available. 

## 4. Discussion

Osteoporosis is a silent disease with non-detectable symptoms, until the occurrence of a fracture, which seriously decreases the quality of life and survival rate in the elderly. Diagnosis of osteoporosis and assessment of fracture risk are based on the analysis of BMD by DXA [32]. However, this measurement only provides information about bone strength. Besides, low BMD is considered a risk factor for fracture, not a disorder marker. The identification of molecules with potential use as biomarkers at the early stages of osteoporosis is a promising research area. 

In this study, through a serum proteomics analysis on a population-based cohort of postmenopausal women, we identified 11 protein spot features that were increased, and 16 that were decreased, in serum from patients with OS and OP relative to the normal samples. A total of 12 serum proteins showed differential expression in the presence of OS and OP. Among the identified proteins showing significant changes in expression between NOR, OS, and OP samples, serum levels of CP, GSN, NADPH 1, and SERPINC 1 were usually lower in osteopenia than in the osteoporosis group. However, the opposite was observed for serum protein levels of KNG1, where higher levels were observed in osteopenia. Of these differentially expressed proteins, VDBP showed the most decreased levels in osteopenia and osteoporosis samples. This validation confirmed that the serum levels of these proteins were altered in women with osteopenia and osteoporosis.

Gelsolin is an actin-binding protein, a member of the superfamily of proteins which are Ca^2+^-dependent [33]. In the bone, GSN participates in the osteoclasts-podosome assembly [34]. To the best of our knowledge, there is only one report showing a negative correlation between plasma GSN levels and hip BMD, in Chinese postmenopausal women [21]. In our proteomic analysis, serum levels of GSN were decreased in the osteopenic and osteoporotic groups. However, when GSN serum levels were assessed by ELISA in a larger sample, the difference between groups did not reach statistical significance. These discrepancies could be related to the type of antibodies used for each assay, sample size, or, more likely, GSN levels could be influenced by other factors.

The physiological function of ceruloplasmin, (CP) includes copper transport, regulation of cellular iron levels, and antioxidant activity by eliminating reactive oxygen species, such as superoxide and hydroxyl radicals [35]. A recent study showed that CP activity was significantly higher in patients with osteoporosis compared to healthy individuals [36]. However, CP levels have never been investigated in osteoporosis patients. These results suggest that CP levels could be playing important roles in BMD loss, and therefore, in the physiopathology of osteoporosis. Additional studies are needed to validate whether serum CP levels may be involved in bone remodeling and eventually lead to the development of osteoporosis.

Vitamin D-binding protein is encoded by the GC gene, it is an alpha-2-globulin partially glycosylated that is produced at the liver and has a molecular weight of 52–59 kDa [37]. The best-recognized function of VDBP is to act as a carrier of vitamin D to reach target tissues for maintaining calcium homeostasis through the vitamin D endocrine system. The VDBP can bind with high affinity to 88% of circulating 25-hydroxyvitamin D (25 (OH) D). Moreover, VDBP is able to bind with low affinity to almost 85% of 1, 25-dihydroxyvitamin D (1, 25 (OH) D), the most active metabolite of vitamin D. The remaining fraction of vitamin D is bound to albumin and <1% of vitamin D exists in an unbound form into the bloodstream [37,38]. In the present study, levels of VDBP were significantly lower in serum from postmenopausal women with low BMD (osteopenia and osteoporosis) and fracture. Our results allowed for establishing a cut-off point to discriminate osteopenic/osteoporotic individuals. Based on these results, serum VDBP could have a potential utility as a biomarker for the early detection of osteopenia and osteoporosis. 

Most studies investigating the effects of VDBP on bone have focused mainly on characterizing VDBP polymorphism on circulating 25(OH)D and attempting to correlate them with bone health [39,40,41]. These studies have investigated three major polymorphic forms of VDBP: GC1F, GC1S, and GC2 (rs7041 and rs4588) [42]. These VDBP variants exhibit differences in affinity for 25(OH)D and 1,25(OH)2D, with the hierarchy of affinity binding GC1F > GC1S > GC2 [43]. On the other hand, experimental evidence has shown that some of these SNPs can influence VDBP serum levels [44,45]. Based on this, altered serum VDPB levels observed in postmenopausal women with low BMD could be influenced by these polymorphisms [46]. Additional studies are needed to evaluate the association between variants in the GC gene, serum VDBP levels, and BMD variation.

These findings reinforce the importance of VDBP as a key player in bone metabolism, especially at the early stage of osteoporosis (osteopenia). Osteopenia is defined as a condition in which BMD is lower than normal, generally detected in younger women and increasing severely with age. The proteomic approach used in the present study provided us with a broader picture of the proteins involved in the pathophysiological process of osteopenia/osteoporosis, leading to the identification of VDBP and other previously described proteins, such as apolipoprotein AI [47], ceruloplasmin, kininogen 1 [47], alpha-2-HS-glycoprotein [28,29], serpin A1, and serpin A3 [30]. Further studies on VDBP are needed to determinate its role in bone remodeling and its importance as an early disease marker. Nonetheless, the protein identified is particularly important because it provides new insight on the variation of BMD and the disease development and may act as an early biomarker, specifically for the Mexican population, which could be related to the ethnic predisposition.

Our study had some limitations to consider. First, the sample size of fracture and osteoporotic postmenopausal women was relatively small, although it is similar to other reports of pilot proteomics studies focused on the search for biomarkers [47,48]. These results should be validated in larger groups of osteoporotic postmenopausal women and other populations. Second, the serum sample amount available was also a limitation (initial discovery stage). An ideal design should have included biological and technical replicates. Due to the lack of samples complying with inclusion and exclusion criteria, we did not analyze individual samples. Instead, we decided to do the initial proteomics analysis performing sample pooling and then, validate the results with an independent cohort of postmenopausal women, on an individual basis through ELISA. Third, the protein coverage was 50%, maybe due to incomplete trypsin digestion, not optimal peptide ionization, and other factors (pH, temperature, and processing of the sample). Trypsin is unable to cleave C-terminal when an arginine or a lysine is directly followed by a proline. Digestion is also less effective for tightly folded proteins and membrane proteins because they possess just a few trypsin cleavage sites. Therefore, it would be necessary to introduce additional proteases to improve the qualitative proteome coverage [49]. This requires further experimental work which is beyond the scope of this study. Perhaps the most important limitation of the present work is the lack of follow-up. However, to validate the diagnosis efficacy of the three identified biomarkers (CP, GSN, and VDBP), a larger cohort study has to be performed. About this, we have plans to conduct future studies with increased sample sizes of patients with osteoporosis and osteoporotic fractures. Additional studies are necessary to analyze if serum VDBP levels in conjunction with the biochemical bone turnover markers (the bone formation marker N-terminal propeptide of type 1 collagen (PINP) and resorption marker carboxy-terminal cross-linking telopeptide of type I collagen (βCTX-I)) and known clinical risk factors could improve the sensitivity and specificity, as an early biomarker of the disease. This analysis will allow us to determine its potential clinical utility to identify the affected individuals at early stages of the disease, in the Mexican population.

Nevertheless, these results could be regarded as preliminary, so that a quantitative proteomics method could be an approach to be used in the future to solve these limitations.

## 5. Conclusions

In summary, the present study suggests that low serum levels of VDBP are associated with low BMD (osteopenia and osteoporosis) and osteoporotic fracture in Mexican postmenopausal women. Here, we propose that serum VDBP has the potential to act as a marker associated with low BMD. VDBP could be a valuable resource for a better diagnosis, prognosis, and management of the disorders affecting BMD. A serum marker would prevent the need for performing a dual-energy X-ray absorptiometry, which implies exposure to radiation and expensive equipment. This finding is an initial step towards the identification of new non-invasive biomarkers. This knowledge will allow the development of new screening strategies for detecting the population at risk of suffering osteoporosis and fragility fractures.

## Figures and Tables

**Table 1:** Demographic characteristics of Mexican women used in the study.

	Proteomic Analysis				ELISA			
Characteristics	Normal NOR (*n* = 10)	Osteopenic OS (*n* = 10)	Osteoporotic OP (*n* = 10)	*p* Value	Normal NOR (*n* = 26)	Osteopenic OS (*n* = 29)	Osteoporotic OP (*n* = 19)	*p* Value
Age (years) *	73 (2)	74 (3)	75 (4)	0.8	65 (8)	67 (7)	73 (9)	<0.001
Weight (kg) *	56 (5)	52 (6)	56 (4)	0.3	63 (12)	62 (12)	54 (8)	0.1
Height (cm) *	153 (5)	151 (4)	148 (6)	0.07	152 (6)	152 (5)	148 (7)	0.09
BMI (kg/m) *	24 (1)	23 (2)	25 (2)	0.8	27 (5)	27 (4)	25 (3)	0.3
Waist circumference (cm) *	90 (6)	88 (8)	94 (7)	0.1	95 (11)	95 (10)	92 (10)	0.5
Body fat proportion *	41 (7)	40 (3)	43 (6)	0.6	44 (6)	45 (6)	40 (8)	0.08
Never smoker, %	80	90	80	0.9	69	66	79	0.9
Smoking Current, %					3.9	14	5.3	
Uric acid (mg/dL) *	5 (2)	5 (1)	6 (1)	0.4	5 (1)	5 (1)	5 (1)	0.5
Systolic blood pressure (mmHg) *	130 (15)	132 (26)	132 (30)	0.8	131 (17)	134 (21)	135 (31)	0.5
Diastolic blood pressure (mmHg) *	71 (13)	72 (12)	71 (10)	1	74 (12)	76 (9)	72 (10)	0.7
Total cholesterol (mg/dL) *	147 (79)	120 (64)	149 (57)	0.6	129 (65)	131 (80)	172 (98)	0.3
Triglycerides (mg/dL) **	172 (110–326)	175 (108–192)	138 (107–177)	0.4	168 (110–277)	129 (102–173)	141 (109–177)	0.07
LDL-C(mg/dL) *	123 (33)	145 (40)	120 (37)	0.9	136 (34)	126 (35)	127 (43)	0.9
HDL-C(mg/dL) *	43 (9)	54(10)	50 (9)	0.04	48 (12)	53 (11)	53 (16)	0.12
Glucose (mg/dL) **	108 (94–150)	98(87–104)	94 (92–103)	0.3	103 (95–129)	99 (89–106)	93 (89–101)	0.048
Hip BMD (g/cm^2^)	0.95 (0.06)	0.78 (0.04)	0.65 (0.03)	<0.001	0.97 (0.03)	0.81 (0.02)	0.68 (0.04)	<0.001
Hip T-score	−0.49 (0.45)	−1.84 (0.32)	−2.83 (0.24)	<0.001	−0.33 (0.25)	−1.59 (0.20)	−2.94 (0.33)	<0.001
Lumbar Spine BMD (g/cm^2^) **	1.35 (0.91, 1.12)	0.87 (0.79, 0.95)	0.79 (0.78, 0.92)	0.03	1.03 (0.92, 1.09)	0.91 (0.87, 1.04)	0.79 (0.74, 0.92)	0.001
Lumbar Spine T-score **	−1.37 (−2.11, −1.01)	−2.59 (−3.36, −1.65)	−3.31 (−3.44, −2.41)	0.02	−1.49 (−2.14, −0.97)	−2.39 (−2.80, −1.38)	−3.40 (−3.74, −2.41)	<0.001

* Mean (SD), ** Median (P25, P75). The differences between groups for continuous variables were analyzed by Analysis of variance (ANOVA) or Dunn test. For the categorical variables tests of proportions were used.

**Table 2:** List of differentially expressed serum proteins identified through pairwise comparison of the groups of postmenopausal women.

Name of Protein	UniProt Accession Number	Gene Name	MW (kDa)	pI	ID Match	Fold Change			*p*-Value (*t*-Test)			Score	(%) Protein Coverage	No. of Matched Peptides
						OS/NOR	OP/NOR	OP/OS	OS/NOR	OP/NOR	OP/OS			
cDNA FLJ56212, highly similar to Gelsolin	B7Z9A0	*GSN*	83.1	5.6	39	−3.3	−1.7	2	0.009	0.5	0.4	4.8	10	2
Carbonyl reductase (NADPH) 1	E9PQ63	*CBR1*	19	5.9	329	−2.7	−1.1	2.45	0.01	0.07	0.7	1.3	12	1
Ceruloplasmin	Q1L857	*CP*	115.5	5.4	132	−2.3	−1.7	1.3	0.06	0.002	0.1	7.3	17	4
Ceruloplasmin	Q1L857	*CP*	115.5	5.4	127	−1.9	−1.3	1.5	0.07	0.01	0.3	3	5.8	1
Serpin peptidase inhibitor, clade C (Antithrombin), member 1, isoform CRA_a	A0A024R944	*SERPINC1*	52.6	6.3	171	−1.5	−1.5	−1.0	0.048	0.7	0.04	12.3	31	10
Ceruloplasmin	Q1L857	*CP*	115.5	5.4	125	−1.6	−1.2	1.3	0.05	0.05	0.2	8	8.9	4
Epididymis secretory protein Li 51/vitamin D-binding protein	V9HWI6	*HEL-S-51/VDBP*	53	5.4	195	−1.4	−2.6	−1.8	0.15	0.02	0.02	16.5	49	10
Kininogen 1, isoform CRA_a	D3DNU8	*KNG1*	47.9	6.3	148	2.2	1.6	−1.3	0.003	0.5	0.5	23.3	44	15

MW: Molecular Weight, kDa: kilodalton, pI: Isoelectric point.

## Supplementary Materials

The following are available online at https://www.mdpi.com/2072-6643/11/12/2853/s1, Supplementary Material: Study Participants;High-Abundant Protein Depletion in Serum; Preparation and Separation of Proteins by 2D-DIGE; Image Acquisition and DIGE Analysis; Protein Spot Picking and in-Gel Digestion; MALDI TOF/TOF; Database Searching. Table S1: Demographic characteristics of Mexican women included in the analysis of VDBP concentration by ELISA (HWCS, *n* = 425, and Fracture, *n* = 21). Table S2: Samples women labeling for 2D-DIGE gels. Table S3: Differentially expressed serum proteins in Mexican postmenopausal women with Osteopenia and Osteoporosis. Table S4: Peptides identification. Figure S1. Depletion of albumin and IgG’s in serum samples. (a) Representative chromatogram obtained after depletion. Fraction A: Depleted proteins and Fraction B: Immunoglobulin G and albumin. (b) SDS-PAGE of serum proteins before and after depletion. Line 1: Weight marker, lane 2: serum before depletion, lane 3: depleted proteins (fraction A), Lane 4: albumin and IgG’s (fraction B). Twenty micrograms of proteins were loaded per lane and separated in 10% polyacrylamide gel and visualized by Coomassie blue staining. Figure S2: Additional differentially expressed proteins in postmenopausal women implicated in bone metabolism. The 3D images show the intensity representation of those proteins selected for their association to BMD and with a role in bone remodeling. Proteins identified in the biological variation analysis (BVA), with *p*-value > 0.05 between the group of women with OS and OP.
