# Supplementary material for: Serum Proteomic Analysis Reveals Vitamin D-Binding Protein (VDBP) as a Potential Biomarker for Low Bone Mineral Density in Mexican Postmenopausal Women
Source: Nutrients. 2019 Nov 21;11(12):2853. doi: 10.3390/nu11122853 (PMC6950314; doi:10.3390/nu11122853)
Supplement: Supplementary file 1 [file nutrients-11-02853-s001.zip › nutrients-629204 SM/Figure S1 and S2.pdf]

a)

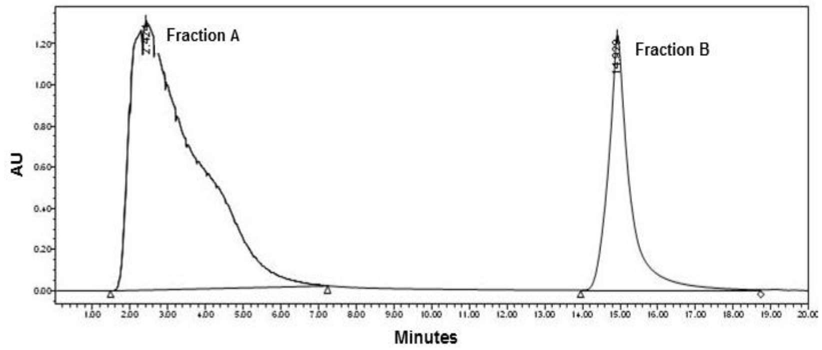

Fraction	Retention time	Area	Area %
A (sample depleted)	2.424	15825585	76.03
B (albumin and IgG's)	14.929	49269392	23.97

b)

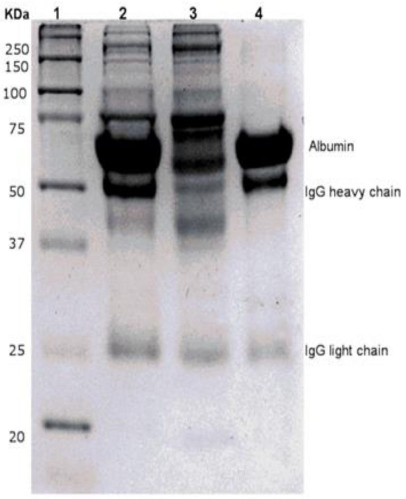

Figure S1: Depletion of albumin and IgG's in serum samples. (**a**) Representative chromatogram obtained after depletion. Fraction A: Depleted proteins and Fraction B: Immunoglobulin G and albumin. (**b**) SDS-PAGE of serum proteins before and after depletion. Line 1: Weight marker, lane 2: serum before depletion, lane 3: depleted proteins (fraction A), Lane 4: albumin and IgG's (fraction B). Twenty micrograms of proteins were loaded per lane and separated in 10% polyacrylamide gel and visualized by Coomassie blue staining.

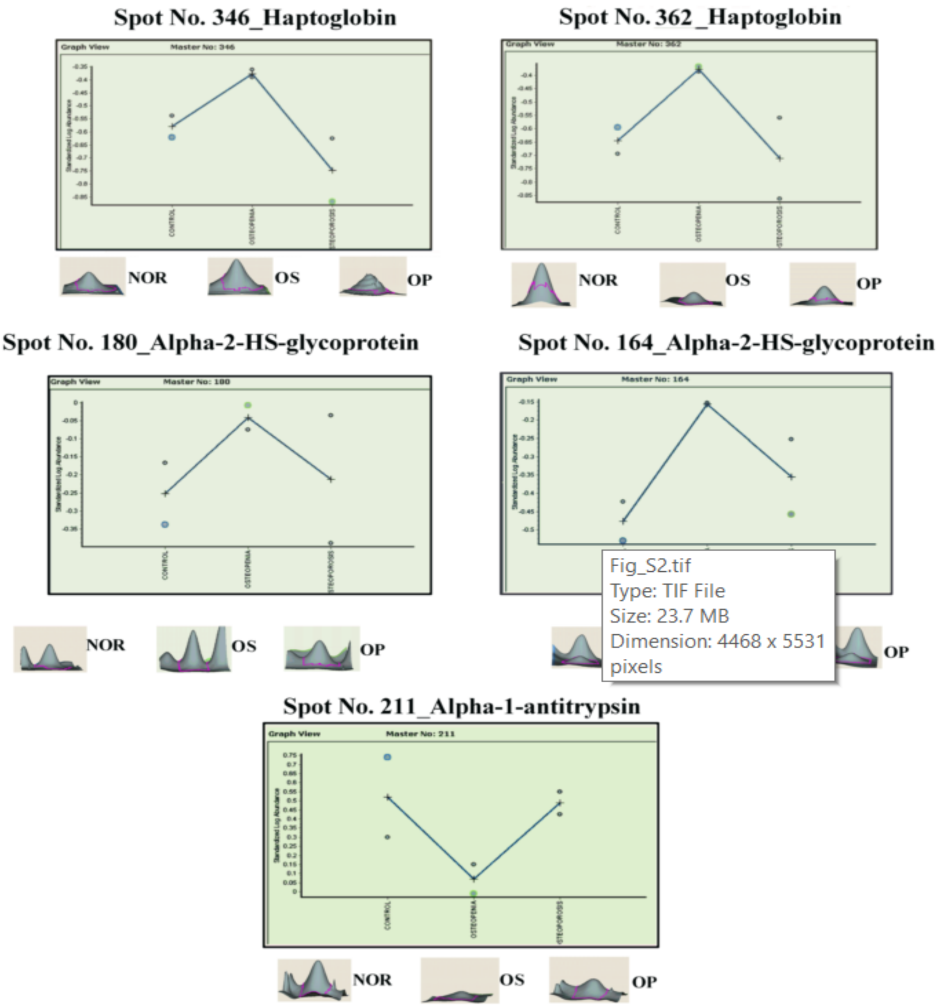

Figure S2: Additional differentially expressed proteins in postmenopausal women implicated in bone metabolism. The 3D images show the intensity representation of those proteins selected for their association to BMD and with a role in bone remodeling. Proteins identified in the biological variation analysis (BVA), with *p*-value > 0.05 between the group of women with OS and OP.
