# Supplementary material for: Serum Proteomic Analysis Reveals Vitamin D-Binding Protein (VDBP) as a Potential Biomarker for Low Bone Mineral Density in Mexican Postmenopausal Women
Source: Nutrients. 2019 Nov 21;11(12):2853. doi: 10.3390/nu11122853 (PMC6950314; doi:10.3390/nu11122853)
Supplement: Supplementary file 1 [file nutrients-11-02853-s001.zip › nutrients-629204 SM/Supplementary material_nutrients_edited.docx]

**Supplementary material**

Study Participants

All women included in this study were from Mexican-Mestizo origin and were recruited from “The Health Workers Cohort Study” (HWCS). Only women born in Mexico whose parents and grandparents identified themselves as Mexican mestizos were included in the study. Additionally, these samples have been genotyped for 96 ancestry informative markers (AIMs) to rule out false associations due to population stratification. These AIMs distinguish mainly Amerindian and European ancestry (δ > 0.44) and have been validated in other study in Mexican population [1]. Overall, the gene pool of the study sample showed 57% Native Amerindian, 35% European, and 7% African component, which is similar to previous reports in mestizos from the state of Guerrero [2].

High-Abundant Protein Depletion in Serum

Human serum was depleted using the albumin and IgG cartridge (Qiagen, Hilden, North Rhine-Westphalia, Germany) according to manufacturer’s instructions on HPLC equipment (Waters Alliance, 2695). Briefly, each sample was diluted 1:3 with PBS (50 mM NaH2PO4; 150 mM NaCl, pH 7.2) and centrifuged at 10,000× *g* at 4 °C for 2 min, the supernatant was filtered through a 0.22 μm membrane. The column was equilibrated with 10 ml of PBS with a flow rate of 1 mL/min at room temperature. Afterwards, the sample was injected into the depletion column with an injection volume of 100 µl, at 10 °C. The unbound serum proteins (Fraction A) were eluted with PBS at a flow rate at 0.2 mL/min, and the fraction was collected from min 2 to min 6. To elute the albumin and IgG, a linear gradient with 100% of 20 mM glycine, pH 2.0 was run for 18 min. The column was washed until the A280 returned to the baseline value. The Fraction A was concentrate and desalted by ultrafiltration using a 3 kDa cut off centrifugal filter device (Millipore, Billerica, Massachusetts, USA). The samples were displaced with to solution Tris 0.5 mM.

Preparation and Separation of Proteins by 2D-DIGE

10 samples from each group (Normal, Osteopenia and Osteoporosis) were selected for the 2D-DIGE analysis. We made two pools for each condition, each pool contained equal protein amounts from five samples. Pools were labeled according to the CyDye DIGE minimal protocol (GE Healthcare, Chicago, Illinois, USA). 50 µg of proteins were taken from each pool and labeled with 400 pmol of Cy3 or Cy5 dye. In addition, an internal standard containing an equal amount of protein was labeled with Cy2 dye. The internal standard was run on all the DIGE gels to assess the reproducibility and minimize the gel-to-gel variation. The labeled samples were mixed and the final quantity was 150 µg of protein per gel, (Supplementary table 2). We added to pooled sample of each gel, 2% *v/v* IPG buffer pH 4–7 and 20 mM DTT and incubated on ice for 10 min prior to bringing the total sample to a final volume of 360 µL with DeStreak buffer (GE Healthcare, Chicago, Illinois, USA). Immobiline Drystrip of 24 cm, pH 4–7 (Bio-Rad, Hercules, California, U.S.A.) was rehydrated overnight at room temperature. First-Dimension Isoelectric Focusing (IEF) was using an Ettan IPGphor 3 system (GE Healthcare, Chicago, Illinois, USA) with the current limit of 50 μA and following this program: first step for 100V/100Vh, second Step for 500V/500Vh, third Grad for 1000V/800Vh, fourth Grad for 10000 V/16500 Vh and fifth Step 10000 V/34700 Vh. After IEF, the strips were equilibrated first with 80 mM DTT (Dithiothreitol) and after 169 mM Iodoacetamide, every equilibration was of 15 min. Second dimension separation was conducted in 12% acrylamide gels cast on an Ettan DALT-Twelve electrophoresis system (GE Healthcare, Chicago, Illinois, USA) at 25 °C and the run consisted of 60 V/1 h and 100 V/16 h.

Image Acquisition and DIGE Analysis

The DIGE gels were scanned using an Ettan DIGE Imager instrument (GE Healthcare, Chicago, Illinois, USA). Cy2 images were scanned with 488 nm Blue2 laser and 520 BP 40 emission filter, Cy3 images were scanned with 532 nm Green laser and 520 BP 30 emission filter and Cy5 images were scanned with 633 nm Red laser and 560 BP 30 emission filter. For differential analysis, the images were normalized and analyzed using the DeCyder 2D software, version 6.5 (GE Healthcare, Chicago, Illinois, USA). The differential in-gel analysis (DIA) module was used to perform intra-gel matching on a merged image from an in-gel set of images. Biological variation analysis (BVA) module was used for inter-gel matching that used the DIA datasets from all individual gels, to found the significant changes in expression of specific proteins between experimental groups. The gel with the highest spot count was assigned as the master gel. Matching between gels was performed utilizing the master gel for each image pair. Landmarking and manually confirming potential spots of interest further improved matching. Student’s t-test was used to identify spots with statistical significant expression. Statistical significance was associated with those differentially expressed spots whose expression has change +1.5 to -1.5 fold at the 95% confidence level within the group. We consider the spots with p-value of ≤0.05 in osteopenic and osteoporotic group versus normal women.

Protein Spot Picking and in-Gel Digestion

Five preparative gels were performed in Immobiline Drystrip of 24 cm, pH 4–7 with 800 µg of protein per gels and stained with Coomassie blue R-250. The spots of interest were manually excised and sent for their identification by mass spectrometry. Spots were detained repeatedly with 50 mM ammonium bicarbonate (NH4HCO3) and 50% acetonitrile (ACN) for 3 times, for 10 min to 50 °C each. Spots were dried at temperature ambient for 5 min with 100% ACN for 5 min. For protein digestion, 20 μg/μL trypsin solution (Trypsin Gol, promega) was added and incubated at 4 °C for 1h and incubated the suspension overnight at 37 °C. Peptides were extracted with 40 mM NH4HCO3 and 10% ACN and overnight incubation at 37 °C. Peptides were centrifuged and the extract was reconstituted with 5% formic acid solution and 50% ACN. Peptides were desalted, concentrated and purified using Zip Tip pipette tip contained C18 resin (Millipore, Billerica, Massachusetts, USA). Peptides were eluted of the ZipTip pipette with 50% ACN in 0.1% Trifluoroacetic acid (TFA) (Sigma-Aldrich, St. Louis, MO, USA).

MALDI TOF/TOF

Peptides were performed with 4800 MALDI-TOF/TOF Analyzer (Applied Biosystems/AB sciex, Waltham, Massachusetts, USA). It was equipped with Nd:YAG 355nm laser and a 4000 Series Explorer software, version 3.5 (with MALDI 4800). The MALDI-TOF/TOF was operated in a positive ion mode and externally calibrated using a mass calibration standard kit (ABSciex, MA, USA), by monitor results in mass range of 900–3600 Da. The laser power was set between 2800 and 2800 for MS and between 3500–3800 for MS/MS acquisition. After, sample were screened in to MS-positive reflector mode using 25 laser shots, the fragmentation of automatically selected precursors was performed at a collision energy of 2 kV using air as collision gas (pressure of 2 × 106 Torr) with an accumulation of 400 shots for each spectrum. MS spectra were acquired between m/z 800 - 4000. For internal calibration, we used the parent ion of Glu-1 fibrino-peptide B at m/z 1570.677 Da diluted in the matrix (1.3 pmol/µL/spot). Up to 16 of the most intense signal per spot position having a S/N of >20 were selected as precursors for MS/MS acquisition.

Database Searching

Peptide identification was performed by the ProteinPilot software version 2.0 (AB Sciex, MA, USA,) using the Paragon algorithm as the search engine. Each MS/MS spectrum was searched for Homo sapiens species against the Uniprot database. Other parameters such as tryptic cleavage specificity, precursor ion mass accuracy and fragment ion mass accuracy are MALDI 4800 built-in functions of ProteinPilot software. The detected protein threshold in the software was set to 2 to achieve >60% confidence, and identified proteins were grouped by the ProGroup algorithm (ABsciex, MA, USA) to minimize redundancy.

Reference

1. Kosoy R.; Nassir R.; Tian C.; White P.A.; Butler L.M.; Silva G.; Kittles, R.; Alarcon‐Riquelme, M.E. Gregersen, P.K.; Belmont, J.W.; et al. Ancestry informative marker sets for determining continental origin and admixture proportions in common populations in America. *Hum Mutat.* **2009**, *30*, 69–78

2. Silva-Zolezzi, I.; Hidalgo-Miranda, A.; Estrada-Gil, J.; Fernandez-Lopez, J.C.; Uribe-Figueroa, L.; Contreras, A.; Balam-Ortizet, E.; Bosque-Plata, L.d.; Velazquez-Fernandez, D.; Lara, C.; et al. Analysis of genomic diversity in Mexican Mestizo populations to develop genomic medicine in Mexico. *Proc Natl Acad Sci U S A* **2009**, *106*, 8611–8616
