# Supplementary material for: Serum Proteomic Analysis Reveals Vitamin D-Binding Protein (VDBP) as a Potential Biomarker for Low Bone Mineral Density in Mexican Postmenopausal Women
Source: Nutrients. 2019 Nov 21;11(12):2853. doi: 10.3390/nu11122853 (PMC6950314; doi:10.3390/nu11122853)
Supplement: Supplementary file 1 [file nutrients-11-02853-s001.zip › nutrients-629204 SM/Supplementary table 1.docx]

**Supplementary table 1**. Demographic characteristics of Mexican women included in the analysis of VDBP concentration by ELISA (HWCS, *n* = 425 and Fracture, *n* = 21).

| **Characteristics** | **Normal NOR** (*n* = 260) | **Osteopenia OS** (*n* = 140) | **Osteoporosis OP** (*n* = 25) | **Fracture** (*n* = 21) |
| --- | --- | --- | --- | --- |
| Age (years)* | 59.5(7.7) | 66.5(8.6) (N vs OS *p* ≤ 0.001) | 74.8(9.5), (N vs OP *p* ≤ 0.001), (OS vs OP *p* = 0.028) | 83.9 (8.4), (N vs F *p* ≤ 0.001), (OP vs F *p* ≤ 0.001) ,(OS vs F *p* ≤ 0.001) |
| Weight (kg)* | 68.2(12.2) | 62.4(10.1) (N vs OS *p* = 0.001) | 53.8(8.1), (N vs OP *p* ≤ 0.001), (OS vs OP *p* = 0.048) | 56.3(9.9), (N vs F *p* ≤ 0.001) |
| Height (cm)* | 153.3(5.7) | 152.6(5.8) | 149.5(7.2), (N vs OP *p* = 0.023) | 1.50(0.05), (N vs F *p* ≤ 0.001), (OP vs F *p* ≤ 0.001), (OS vs F *p* ≤ 0.001) |
| BMI (kg/m)* | 29.0(5.0) | 26.8(4.1) | 24.1(3.0), (N vs OP *p* = 0.002) | 24.8(3.5) (N vs F *p* = 0.010) |
| Waist circumference (cm)* | 97.0(11.3) | 94.6(11.0) | 90.8(9.6), (N vs OP *p* = 0.027) |  |
| Body fat proportion* | 46.7(6.1) | 44.4(5.9) (N vs OS *p* = 0.002) | 39.2(7.2), (N vs OP *p* ≤ 0.001), (OP vs OP *p* = 0.001) |  |
| Never smoker,% | 63.1 | 61.4 | 76.0 |  |
| Smoking  Current,% | 5.0 | 10.0 | 4.0 |  |
| Past | 27.3 | 22.1 | 12.0 |  |
| Uric acid (mg/dL)* | 5.4(1.2) | 5.1(1.1) (N vs OS *p* = 0.042) | 5.2(1.3) |  |
| Systolic blood pressure (mmHg)* | 123.7(16.9) | 129.0(18.5) (N vs OS *p* = 0.018) | 132.1(28.8) |  |
| Diastolic blood pressure (mmHg)* | 73.7(11.3) | 72.3(9.7) | 70.4(10.1) |  |
| Creatinine(mg/dL)** | 0.78(0.67-0.90) | 0.80(0.70-0.90) | 0.80(0.69-0.89) |  |
| Total cholesterol (mg/dL)* | 145.6(103.1) | 152.5(121.9) | 160.9(91.1) |  |
| Triglyrecide (mg/dL)** | 164(125-218) | 156(115-196) (N vs OS *p* = 0.018) | 141(110-167), (N vs OP *p* = 0.017) |  |
| LDL-C(mg/dL)* | 134.2(37.8) | 135.2(42.9) | 128.0(39.1) |  |
| HDL-C(mg/dL)* | 44.4(10.6) | 49.1(13.1) (N vs OS *p* ≤ 0.001) | 52.4(15.1), (N vs OP *p* = 0.004) |  |
| Glucose (mg/dL)** | 99(93-108) | 97(90-105) (N vs OS *p* = 0.009) | 93(89-100), (N vs OP *p* = 0.004) |  |
| Bone Mineral Density (g/cm2) | 1.00(0.09) | 0.81(0.05) (N vs OS *p* ≤ 0.001) | 0.63(0.06), (N vs OP *p* ≤ 0.001), (OP vs OS *p* ≤ 0.001) |  |
| T-score (Hip) | -0.02(0.72) | -1.54(0.39) (N vs OS *p* ≤ 0.001) | -3.01(0.47), (N vs OS *p* ≤ 0.001), (OS vs OP *p* ≤ 0.001) |  |
| Femoral neck (g/cm2)* | 0.95(0.09) | 0.78(0.07) (N vs OS *p* ≤ 0.001) | 0.64(0.08), (N vs OP *p* ≤ 0.001), (OS vs OP *p* ≤ 0.001) |  |
| Spine BMD (g/cm2)** | 1.04(0.96-1.13) | 0.92(0.84-1.02), (N vs OS *p* ≤ 0.001) | 0.79(0.74-0.92), (N vs OP *p* ≤ 0.001), (OS vs OP *p* = 0.003) |  |

*Mean (SD). **Median (P25-P75). The differences between groups for continuous variables were analyzed by ANOVA or Dunn test. For the categorical variables tests of proportions was used.
