# Supplementary material for: Serum Proteomic Analysis Reveals Vitamin D-Binding Protein (VDBP) as a Potential Biomarker for Low Bone Mineral Density in Mexican Postmenopausal Women
Source: Nutrients. 2019 Nov 21;11(12):2853. doi: 10.3390/nu11122853 (PMC6950314; doi:10.3390/nu11122853)
Supplement: Supplementary file 1 [file nutrients-11-02853-s001.zip › nutrients-629204 SM/Supplementary table 2.docx]

**Supplementary table 2.** Samples women labeling for 2D-DIGE gels.

| Experiment dissent | | | |
| --- | --- | --- | --- |
| No. GEL | **Cy3 (50µg)** | **Cy5 (50µg)** | **Cy2 (50µg)** |
| 1 | Normal | osteoporosis | Internal standard |
| 2 | Normal | osteopenia | Internal standard |
| 3 | osteoporosis | osteopenia | Internal standard |
