# Supplementary material for: Serum Proteomic Analysis Reveals Vitamin D-Binding Protein (VDBP) as a Potential Biomarker for Low Bone Mineral Density in Mexican Postmenopausal Women
Source: Nutrients. 2019 Nov 21;11(12):2853. doi: 10.3390/nu11122853 (PMC6950314; doi:10.3390/nu11122853)
Supplement: Supplementary file 1 [file nutrients-11-02853-s001.zip › nutrients-629204 SM/Supplementary table 3.doc]

**Supplementary Table 3**. Differentially expressed serum proteins in Mexican postmenopausal women with Osteopenia and Osteoporosis.

| **No** | | **Name of protein** | **UniProt accession number** | |  | | **Gene name** | **MW(kDa)** | **pI** | **ID Match** | **Fold Change** | | | ***p* value (*t*-test)** | | | **Score** | **(%) Protein coverage** | **No. of matched peptides** | |
| --- | --- | --- | --- | --- | --- | --- | --- | --- | --- | --- | --- | --- | --- | --- | --- | --- | --- | --- | --- | --- |
|  |  |  |  |  |  |  |  |  |  |  | **OS/N** | **OP/N** | **OP/OS** | **OS/N** | **OP/N** | **OP/OS** |  |  |  |  |
| **Down-regulated protein** | | | | | | | | | | | | | | | | | | | | |
| **1** | | cDNA FLJ56212, highly similar to Gelsolin | B7Z9A0 | |  | | GSN | 83.103 | 5.58 | **39** | −3.25 | −1.67 | 1.96 | 0.0086 | 0.48 | 0.38 | 4.8 | 10.1 | 2 | |
| **2** | | Apolipoprotein A-I, isoform CRA_a | A0A024R3E3 | |  | | APOA1 | 30.77 | 5.56 | **320** | −3.25 | −1.58 | 2.06 | 0.052 | 0.37 | 0.44 | 22 | 67.4 | 11 | |
| **3** | | Carbonyl reductase [NADPH] 1 | E9PQ63 | |  | | CBR1 | 19.02 | 5.87 | **329** | −2.7 | −1.1 | 2.45 | 0.013 | 0.067 | 0.69 | 1.3 | 11.8 | 1 | |
| **4** | | Alpha-1-antitrypsin | A0A0G2JRN3 | |  | | SERPINA1 | 40.234 | 5.26 | **211** | −2.72 | 1.02 | 2.77 | 0.22 | 0.088 | 0.89 | 21.7 | 56.8 | 10 | |
| **5** | | Apolipoprotein A-I, isoform CRA_a | A0A024R3E3 | |  | | APOA1 | 30.77 | 5.56 | **328** | −2.52 | −1.06 | 2.38 | 0.055 | 0.063 | 0.49 | 10.13 | 41.6 | 6 | |
| **6** | | Apolipoprotein A-I, isoform CRA_a | A0A024R3E3 | |  | | APOA1 | 30.77 | 5.56 | **325** | −2.54 | −1.37 | 1.86 | 0.13 | 0.32 | 0.58 | 20.7 | 53.2 | 10 | |
| **7** | | Ceruloplasmin | Q1L857 | |  | | CP | 115.47 | 5.43 | **132** | −2.26 | −1.74 | 1.29 | 0.059 | 0.0017 | 0.12 | 7.3 | 16.8 | 4 | |
| **8** | | Apolipoprotein A-I, isoform CRA_a | A0A024R3E3 | |  | | APOA1 | 30.77 | 5.56 | **330** | −2.08 | −1.6 | 1.3 |  | 0.37 |  | 19.5 | 69.3 | 9 | |
| **9** | | Epididymis secretory sperm binding protein Li 62p | V9HWA9 | |  | | HEL-S-62p | 187.14 | 6.02 | **262** | −1.91 | −1.75 | 1.09 | 0.21 | 0.68 | 0.27 | 21.3 | 24.1 | 11 | |
| **10** | | Ceruloplasmin | Q1L857 | |  | | CP | 115.47 | 5.43 | **127** | −1.85 | −1.27 | 1.46 | 0.072 | 0.014 | 0.34 | 2.96 | 5.8 | 1 | |
| **11** | | Haptoglobin | P00738 | |  | | HP | 45.2 | 6.13 | **359** | −1.82 | −1.48 | 1.23 |  |  |  | 20.3 | 36.7 | 10 | |
| **12** | | Epididymis secretory sperm binding protein Li 62p | V9HWA9 | |  | | HEL-S-62p | 187.14 | 6.02 | **258** | −1.73 | −1.65 | 1.05 | 0.16 | 0.95 | 0.28 | 10.1 | 10 | 6 | |
| **13** | | Serpin peptidase inhibitor, clade C (Antithrombin), member 1, isoform CRA_a | A0A024R944 | |  | | SERPINC1 | 5260 | 6.32 | **171** | −1.47 | −1.51 | −1.03 | 0.048 | 0.68 | 0.045 | 12.34 | 31 | 10 | |
| **14** | | Ceruloplasmin | Q1L857 | |  | | CP | 115.47 | 5.43 | **125** | −1.57 | −1.21 | 1.29 | 0.052 | 0.05 | 0.2 | 8.02 | 8.9 | 4 | |
| **15** | | Epididymis secretory protein Li 51/ Vitamin D binding protein | V9HWI6 | |  | | HEL-S-51 / VDBP | 52.963 | 5.4 | **195** | −1.43 | −2.57 | −1.8 | 0.15 | 0.022 | 0.019 | 16.5 | 48.9 | 10 | |
| **16** | | Haptoglobin | P00738 | |  | | HP | 45.2 | 6.13 | **363** | −1.15 | 1.08 | 1.24 |  |  |  | 16.9 | 38.2 | 8 | |
| **Up-regulated protein** | | | | | | | | | | | | | | | | | | | | |
| **1** | cDNA FLJ55606, highly similar to Alpha-2-HS-glycoprotein | | B7Z8Q2 |  | | N/A | | 46.62 | 5.84 | **245** | 2.27 | −1.04 | −2.35 | 0.42 | 0.42 | 0.91 | 13 | 41.6 | 8 |  |
| **2** | Kininogen 1, isoform CRA_a | | D3DNU8 |  | | KNG1 | | 47.85 | 6.29 | **148** | 2.19 | 1.63 | −1.34 | 0.0031 | 0.48 | 0.48 | 23.3 | 43.8 | 15 |  |
| **3** | Epididymis secretory sperm binding protein Li 62p | | V9HWA9 |  | | HEL-S-62p | | 187.14 | 6.02 | **232** | 2.08, | 2.04 | −1.02 |  |  |  | 12.6 | 24.2 | 7 |  |
| **4** | highly similar to Alpha-2-HS-glycoprotein | | B7Z556 |  | | N/A | | 27.35 | 6.07 | **169** | 2.01 | 1.34 | −1.5 | 0.072 | 0.27 | 0.51 | 6.85 | 24.5 | 3 |  |
| **5** | Haptoglobin | | P00738 |  | | HP | | 45.2 | 6.13 | **345** | 1.85 | 2.59 | 1.4 |  |  |  | 15.4 | 37.7 | 7 |  |
| **6** | Alpha-2-HS-glycoprotein | | C9JV77 |  | | AHSG | | 39.41 | 5.43 | **164** | 1.84 | 1.2 | −1.54 |  |  |  | 3.52 | 12.2 | 2 |  |
| **7** | Apolipoprotein A-I, isoform CRA_a | | A0A024R3E3 |  | | APOA1 | | 30.77 | 5.56 | **348** | 1.63 | 1.32 | −1.24 |  |  |  | 12.6 | 46.1 | 6 |  |
| **8** | Alpha-2-HS-glycoprotein | | C9JV77 |  | | AHSG | | 39.41 | 5.43 | **180** | 1.6 | 1.17 | −1.37 | 0.15 | 0.44 | 0.86 | 14 | 39.7 | 9 |  |
| **9** | Haptoglobin | | J3QR68 |  | | HP | | 45.04 | 6.13 | **229** | 1.6 | 1.29 | −1.24 | 0.14 | 0.51 | 0.54 | 24.3 | 60.1 | 11 |  |
| **10** | Haptoglobin | | J3QR68 |  | | HP | | 45.04 | 6.13 | **231** | 1.38 | 1.22 | −1.13 | 0.41 | 0.64 | 0.68 | 23 | 51.7 | 13 |  |
| **11** | cDNA FLJ35730 fis, clone TESTI2003131, highly similar to alpha-1-antichymotrypsin | | B3KS79 |  | | N/A | | 5062 | 5.42 | **130** | 1.25 | −1.01 | −1.26 | 0.05 | 0.059 | 0.87 | 14.52 | 30.8 | 10 |  |
